# Supplementary material for: End Sequence Analysis Toolkit (ESAT) expands the extractable information from single-cell RNA-seq data
Source: Genome Res. 2016 Oct;26(10):1397–410. doi: 10.1101/gr.207902.116 (PMC5052061; doi:10.1101/gr.207902.116)
Supplement: Supplemental Material [file supp_gr.207902.116_Supp_Materials.tar.gz › ESAT-esat_v0.1/doc/html/classbroad_1_1pda_1_1geneexpression_1_1gene_exp_utils.html]

ESAT: broad.pda.geneexpression.geneExpUtils Class Reference


ESAT


- Main Page
- Related Pages
- Packages
- Classes
- Files

- Class List
- Class Index
- Class Hierarchy
- Class Members

All Classes Namespaces Files Functions Variables Enumerator Pages

- broad
- pda
- geneexpression
- geneExpUtils

Static Public Member Functions |
List of all members

broad.pda.geneexpression.geneExpUtils Class Reference

|  |  |
| --- | --- |
| Static Public Member Functions | |
| static void | main (String[] args) throws Exception |
|  | |

## Member Function Documentation

|  |  |  |  |  |  |
| --- | --- | --- | --- | --- | --- |
| static void broad.pda.geneexpression.geneExpUtils.main | ( | String[] | *args* | ) | throws Exception |

static

---

The documentation for this class was generated from the following file:

- java/broad/pda/geneexpression/geneExpUtils.java


---

Generated on Fri Aug 15 2014 15:11:00 for ESAT by  

 1.8.7
